# Plasmalogen. Quo vadis?

**DOI:** 10.1016/j.jlr.2025.100814

**Published:** 2025-04-23

**Authors:** Katrin Watschinger

**Affiliations:** Institute of Molecular Biochemistry, Biocenter, Medical University of Innsbruck, Innsbruck, Austria

In recent years, there has been an upsurge in the literature on plasmalogens, with more than 100 publications per year devoted to these distinctive glycerophospholipids that possess a vinyl ether double bond at the *sn-1* position of the glycerol backbone. While the biosynthesis of this particular lipid subclass, starting in the peroxisomes and ending at the endoplasmic reticulum, has been the subject of extensive research, the degradation pathway of these compounds remains to be further elucidated. Plasmalogen breakdown is a complex process involving enzymatic hydrolysis, oxidative cleavage, and possibly also a recycling mechanism. The hydrolysis of plasmalogens by (lyso)plasmalogenases and phospholipases plays an important role in this process (Fig. 1). In the current issue of the *Journal of Lipid Research*, Paul *et al.* contribute to our understanding of plasmalogen degradation by presenting two knockout mouse models for lysoplasmalogenase TMEM86B, one global and one hepatocyte-specific (1). While these mice do not exhibit an obvious systemic phenotype, the lipidome of the liver, plasma, and natural killer cells displayed significantly elevated amounts of plasmalogen, thereby demonstrating the importance of TMEM86B in raising endogenous plasmalogen levels.

**Fig. 1:**
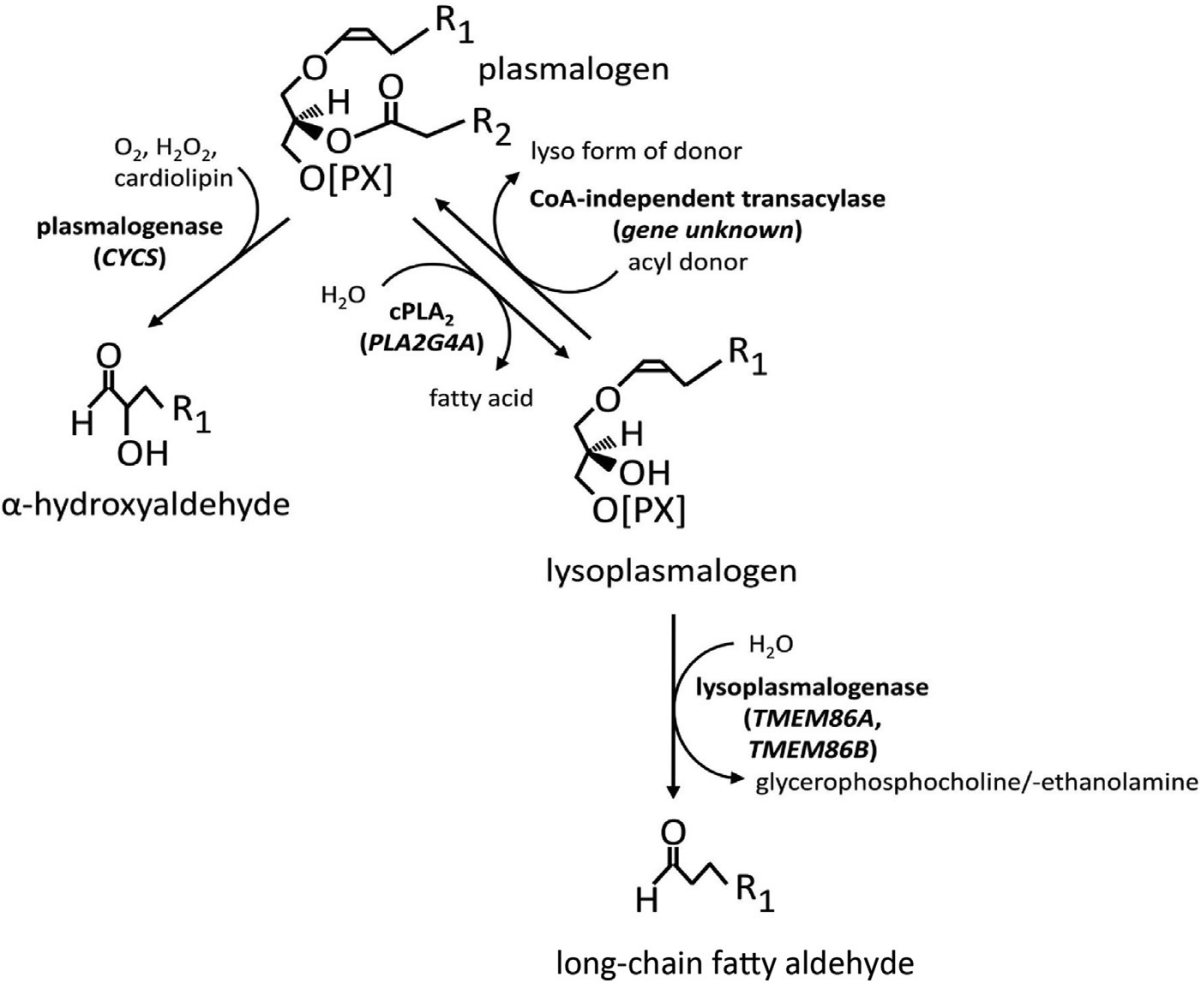
Current knowledge of plasmalogen breakdown by plasmalogenases and lysoplasmalogenases. The plasmalogen bond can be cleaved directly by plasmalogenase cytochrome c (gene symbol: CYCS) in the presence of molecular oxygen, hydrogen peroxide and cardiolipin to yield an α-hydroxyaldehyde. Alternatively, cytosolic phospholipase A_2_ (cPLA_2_, gene symbol: PLA2G4A) can cleave the sn-2 esterified fatty acid to release lysoplasmalogens, the vinyl ether bond of which is then further hydrolytically cleaved by lysoplasmalogenases TMEM86A and TMEM86B to a long-chain fatty aldehyde. The reacylation of lysoplasmalogens to plasmalogens is catalysed by an enzyme known as CoA-independent transacylase, for which the gene has not yet been identified. R1 and R2 denote hydrocarbon side chains of mammalian lipids, comprising typically 14 or 16 carbon atoms and zero or one double bond (R_1_) and approximately 14–22 carbon atoms and 1–6 double bonds (R_2_).

TMEM86B was identified in 2011 as the gene encoding a lysoplasmalogenase, following the first observations of lysoplasmalogenase activity already in the last century (2). It took until 2022 to show that TMEM86A, a close homolog of TMEM86B, is also a lysoplasmalogenase. This was achieved by analyzing the lipidome of an adipocyte-specific TMEM86A knockout mouse, where higher levels of lysoplasmalogens were found in the adipose tissue, proving that the breakdown of these lipids was prevented in this mouse model (3). In addition to these two enzymes, cytochrome c (gene symbol: *CYCS*), a prominent mitochondrial enzyme of the electron transport chain and apoptosis, was found in 2018 to be involved in plasmalogen breakdown through plasmalogenase activity. This enzyme was shown to cleave the vinyl ether double bond under oxidative conditions to yield an α-hydroxyaldehyde (for review, see (4)).

A fundamental unresolved question in the field of plasmalogen catabolism is which of the two possible reaction routes, either 1) directly via plasmalogenase or 2) via a deacylation step by a plasmalogen-specific phospholipase A2 (cPLA2, *PLA2G4A*), yielding a lysoplasmalogen as the first degradation product (5) and subsequent hydrolysis of the ether bond by a lysoplasmalogenase such as TMEM86A and TMEM86B, is actually the more important one (Fig. 1). It is also unclear how these pathways interact or compensate for each other, how they are regulated and whether they are tissue- or cell type–specific. To make the story even more complex, a CoA-independent transacylase activity was described that reacylates lysoplasmalogen intermediates back to plasmalogens by transferring polyunsaturated fatty acids to the vacant *sn-2* position of ether lysophospholipids (Fig. 1), but no gene for this enzyme has so far been identified (6).

But why is plasmalogen breakdown so important? Disturbances in plasmalogen metabolism are associated with several human disorders. Neurodegenerative diseases such as Alzheimer's disease, Parkinson's disease, and multiple sclerosis have been shown to be associated with reduced levels of plasmalogens. Of particular concern are rare inherited syndromes such as peroxisomal biogenesis disorders of the Zellweger spectrum and the monogenic disorder Rhizomelic chondrodysplasia punctata, which are both characterized by loss of plasmalogens from birth (7). Zellweger patients are more severely affected because, in addition to ether lipid metabolism, other peroxisome-based functions such as specific fatty acid catabolism, hydrogen peroxide generation and scavenging cannot be performed due to the lack of functional peroxisome assembly in this disorder, leading to early death in affected children. In Rhizomelic chondrodysplasia punctata, the synthesis of plasmanyl lipids and plasmalogens is specifically disrupted by defects in early peroxisomal enzymes of the ether lipid pathway, leaving the other mentioned peroxisomal pathways unaffected. Patients here present with a distinctive bone and eye phenotype, distinctive facial features, intellectual disability, heart defects, and respiratory problems and may live beyond the first decade in milder forms (7). In both disorders, however, plasmalogen depletion may exacerbate the disease. Knowing how to regulate the metabolism would be a beneficial strategy to benefit both young and old patients with residual plasmalogen biosynthetic capacity but has been inaccessible due to our limited knowledge.

One way to gain some insight into the effect, importance, and regulation of proteins involved in a particular metabolic reaction is to analyze their organelle and tissue expression patterns. What we know so far is that TMEM86A has several transmembrane domains and has been shown to localize to the endoplasmic reticulum (3). According to the Human Protein Atlas, NCBI gene, and the Genotype-Tissue Expression project (www.proteinatlas.org, www.ncbi.nlm.nih.gov/gene/, https://gtexportal.org), its gene expression is highest in skin, kidney, ovary, and brain. TMEM86B is also a transmembrane protein with potential association with the endoplasmic reticulum and Golgi apparatus and is predominantly found in the gallbladder and liver, followed by the intestine, lymph nodes and bone marrow, and brain. In contrast to the two proteins TMEM86A and TMEM86B, cytochrome c is much better studied. It is associated with the inner mitochondrial membrane and can be released into the cytosol during apoptosis. It has a wide tissue distribution with most abundant gene expression levels in the digestive tract and heart. Unfortunately, it is still too early to draw conclusions about the individual roles of TMEM86A and TMEM86B, as their cellular localisation and function are not sufficiently studied, and reliable antibodies for these proteins are not yet available. The localization of the two TMEM86 homologs overlaps to some extent, as shown, for example, by their gene expression in small intestine (1). However, whether one isoform is able to compensate for a deficiency in the other is uncertain and was not found in small intestine of *Tmem86b* knockout mice (1). According to the authors, the reason for this might be that the actual levels of Tmem86a were sufficient to maintain adequate lysoplasmalogenase activity. Another way to increase the knowledge in the field of plasmalogen science is to provide new analytical methods. Here, we recently contributed by publishing a novel HPLC-based assay, which allows direct and fast measurement of lysoplasmalogenase activity (8).

Ether lipid metabolism includes not only plasmalogens, but also non-vinyl ether lipids, the so-called plasmanyl lipids. Analogous to the cleavage by lysoplasmalogenases, alkylglycerol monooxygenase-mediated cleavage of plasmanyl lipids yields aldehydes (9) and these noxious products can cause oxidative damage to cellular components and, more globally, to tissues such as the brain, which is particularly rich in these lipids (4). Long-chain aldehydes are also being discussed as a critical factor in neurodegenerative diseases such as Alzheimer's disease, where oxidative stress and the accumulation of oxidative by-products from plasmalogen degradation may play a role in pathogenesis. In light of this, there is an urgent need to invest in research on plasmalogen degradation. The study by Paul *et al.* in this issue (1) adds another new facet to this chapter.

## Conflict of interest

The author declares that she has no conflicts of interest with the content of this article.
